# The Utilization of Inclisiran for the Optimization of Lipid Management in People Living with HIV: A Clinical Case Series and Comprehensive Review

**DOI:** 10.3390/reports9030284

**Published:** 2026-08-25

**Authors:** Vasileios Petrakis, Maria Panopoulou, Anastasia Grapsa, Andreas G. Tsantes, Periklis Panagopoulos

**Affiliations:** 1 2nd University Department of Internal Medicine, Department of Infectious Diseases, University General Hospital Alexandroupolis, Democritus University Thrace, 68100 Alexandroupolis, Greece; 2 University Laboratory of Microbiology, University General Hospital Alexandroupolis, Democritus University Thrace, 68100 Alexandroupolis, Greece; mpanopou@med.duth.gr; 3 Biopathology Laboratory, University General Hospital Alexandroupolis, Democritus University Thrace, 68100 Alexandroupolis, Greece; 4 Laboratory of Haematology and Blood Bank Unit, “Attiko” Hospital, School of Medicine, National and Kapodistrian University of Athens, 12462 Athens, Greece; 5 Microbiology Department, “Saint Savvas” Oncology Hospital, 11522 Athens, Greece

**Keywords:** inclisiran, HIV infection, dyslipidemia, PCSK9 inhibitors, statin intolerance, atherosclerosis

## Abstract

**Background and Clinical Significance**: People with HIV (PWH) experience an elevated risk of atherosclerotic cardiovascular disease (ASCVD), driven by chronic immune activation, metabolic toxicities of antiretroviral therapy (ART), and traditional risk factors. Achieving target low-density lipoprotein cholesterol (LDL-C) levels is frequently impeded by adherence barriers, pharmacokinetic drug interactions, or muscle-related symptoms. Inclisiran is a hepatocyte-targeted small interfering RNA that halts proprotein convertase subtilisin/kexin type 9 synthesis, providing a long-acting therapeutic alternative. **Case Presentation**: We present two PWH with severe hypercholesterolemia and elevated cardiovascular risk on stable ART. Case 1 describes a 54-year-old male with a history of myocardial infarction presenting with persistent, refractory hypercholesterolemia on rosuvastatin and ezetimibe (baseline LDL-C 142 mg/dL). Case 2 describes a 58-year-old male with verified statin intolerance and inadequate response to ezetimibe (baseline LDL-C 194 mg/dL). Following subcutaneous inclisiran administration at Day 1 and Day 90, Case 1 achieved an 80.2% LDL-C reduction to 28 mg/dL at Month 6, and Case 2 achieved a 54.6% reduction to 88 mg/dL at Month 6 as monotherapy. Both patients tolerated therapy well, with stable CD4+ counts and sustained virological suppression. **Conclusions**: These cases illustrate that inclisiran can effectively lower LDL-C levels across primary and secondary prevention settings in PWH facing oral therapy limitations or statin intolerance. Provider-administered dosing every 6 months overcomes adherence challenges, supporting the inclusion of PWH in broader clinical pathways pending ongoing cardiovascular outcome trials.

## 1. Introduction and Clinical Significance

With the widespread implementation of antiretroviral therapy (ART), human immunodeficiency virus (HIV) infection has transitioned from a uniformly fatal disease into a manageable chronic condition [1]. However, virological suppression does not fully mitigate long-term metabolic and vascular complications [1,2]. People with HIV (PWH) exhibit a significantly elevated risk of cardiovascular disease (CVD), particularly atherosclerotic cardiovascular disease (ASCVD), including myocardial infarction and stroke, which can be up to twice as high as that observed in the general population [2].

The pathogenesis of HIV-associated dyslipidaemia and accelerated atherogenesis is multifactorial, involving a complex interplay between traditional risk factors, direct viral mechanisms, and ART-related metabolic toxicities [3,4]. HIV accessory proteins such as Vpr, Nef, and Tat directly disturb lipid homeostasis: Vpr inhibits peroxisome proliferator-activated receptor gamma (PPAR-gamma), leading to free fatty acid overaccumulation, while Nef impairs cholesterol efflux from infected macrophages by downregulating ATP-binding cassette transporter A1 (ABCA1) function, leading to a profound high-density lipoprotein cholesterol (HDL-C) deficiency [3,4]. Furthermore, chronic low-grade immune activation and residual vascular inflammation persist despite effective virological suppression, fostering the development of subclinical vulnerable plaques [5,6].

Concurrently, several components of traditional and contemporary ART regimens induce or exacerbate dyslipidaemia [7,8,9]. Ritonavir-boosted protease inhibitors (PIs) interact with the pregnane X receptor (PXR) to substantially elevate total cholesterol (TC) and triglycerides (TG), while nucleoside reverse transcriptase inhibitors (NRTIs) like tenofovir alafenamide (TAF) and certain integrase strand transfer inhibitors (INSTIs) are associated with unfavorable lipid shifts and weight gain when compared to lipid-neutral options like tenofovir disoproxil fumarate (TDF) [7,8,9].

The landmark phase 3 REPRIEVE trial established the critical importance of primary cardiovascular prevention in this population, demonstrating that pitavastatin (4 mg daily) reduced major adverse cardiovascular events (MACE) by 35% over a median follow-up of 5.1 years in PWH at low-to-moderate traditional risk [10]. While clinical practice guidelines—including the European AIDS Clinical Society (EACS) and ESC/EAS guidelines—mandate aggressive LDL-C targets for high- and very-high-risk patients, reaching these goals remains challenging [11,12]. Historically, clinical management in PWH has focused predominantly on virological suppression and immunological reconstitution, frequently relegating aggressive ASCVD risk mitigation to secondary importance [12]. Furthermore, maintaining adherence to daily oral statins is compounded by pill fatigue, perceived statin-associated muscle symptoms (driven in substantial part by nocebo effects and public negative bias), and real pharmacokinetic interactions between specific statins and boosted ART regimens [12]. While true statin-induced rhabdomyolysis is exceptionally rare, skeletal symptoms in PWH are frequently multifactorial—arising from HIV-associated myopathy, chronic inflammation, or physical deconditioning—and can be erroneously misattributed to statin toxicity [13]. Interestingly, evidence suggests that statin therapy does not exacerbate muscle wasting and may even preserve muscle mass and function in men living with HIV [13]. Nonetheless, for patients with verified intolerance or severe residual dyslipidaemia on maximally tolerated oral therapy, non-statin alternatives are essential [13].

Targeting proprotein convertase subtilisin/kexin type 9 (PCSK9) has emerged as an indispensable strategy for profound LDL-C lowering. In PWH, monoclonal antibodies (mAbs) targeting circulating PCSK9 (such as evolocumab and alirocumab) have demonstrated safety and robust LDL-C reductions in prospective and registry data, including the BEIJERINCK trial and multicenter cohort analyses [14]. However, mAb regimens require lifelong, self-administered subcutaneous injections every 2 to 4 weeks, which can introduce adherence hurdles in populations already navigating complex oral ART regimens [14]. Inclisiran provides a distinct, highly practical alternative [15]. As a hepatocyte-targeted synthetic small interfering RNA (siRNA) conjugated to triantennary N-acetylgalactosamine (GalNAc), inclisiran undergoes rapid hepatic uptake via asialoglycoprotein receptors and is cleared from the systemic bloodstream within 24 to 48 h [15]. Once internalized, it engages the intracellular RNA-induced silencing complex (RISC) to direct catalytic degradation of PCSK9 mRNA, providing sustained suppression of PCSK9 translation for 6 to 9 months from a single administration [15]. Crucially for people with HIV and complex comorbidities, inclisiran is neither metabolized by cytochrome P450 enzymes nor eliminated via renal transporter pathways, completely eliminating pharmacokinetic drug–drug interactions with ART [15]. While large phase 3 trials confirmed its efficacy across both secondary ASCVD and high-risk primary prevention populations, PWH were not specifically stratified in these pivotal trials. Real-world reports detailing provider-administered inclisiran across distinct primary and secondary prevention settings in PWH remain vital to bridge this gap. This case series evaluates the clinical safety, tolerability, and lipid-lowering efficacy of inclisiran in two complex cases of PWH with severe hypercholesterolemia.

## 2. Case Presentation

### 2.1. Case 1: Advanced Coronary Heart Disease Requiring Intensive Secondary Prevention

A 54-year-old male with a 15-year history of chronic HIV infection presented to our clinic for optimized lipid management. The patient was stably maintained on an antiretroviral therapy (ART) regimen comprising bictegravir, emtricitabine, and tenofovir alafenamide (BIC/FTC/TAF), maintaining an undetectable plasma HIV-1 RNA viral load (<20 copies/mL) and a CD4+ T-cell count of 680 cells/mm^3^. His medical history was notable for a type 1 acute myocardial infarction (ST-elevation myocardial infarction) two years prior, which required emergency percutaneous coronary intervention (PCI) with the deployment of two drug-eluting stents in the left anterior descending coronary artery. A positive family history of premature coronary artery disease suggested a possible underlying polygenic or familial hypercholesterolemia phenotype. Following his acute coronary syndrome, the patient was initiated on a high-intensity oral lipid-lowering regimen consisting of rosuvastatin 20 mg daily combined with ezetimibe 10 mg daily. Despite documented adherence to both his ART and oral lipid-lowering therapy, his lipid panel remained markedly elevated and subtherapeutic according to secondary prevention targets. His baseline lipid profile on maximally tolerated dual oral therapy revealed a total cholesterol of 224 mg/dL, an LDL-C of 142 mg/dL, triglycerides of 158 mg/dL, and an HDL-C of 41 mg/dL (Table 1).

Given his classification as a very-high-risk secondary prevention patient, guideline-directed management mandated an LDL-C target of <55 mg/dL, necessitating an additional >60% reduction. Modifying his ART regimen was not considered appropriate given long-term virological suppression and historical resistance considerations. Oral bempedoic acid was deemed insufficient in potency to achieve this target reduction, while triglyceride-specific therapies (such as icosapent ethyl) were not indicated with a baseline triglyceride level of 158 mg/dL. Due to the patient’s strong preference to avoid biweekly or monthly subcutaneous self-injections, healthcare provider-administered inclisiran was selected as add-on therapy. The patient received an initial subcutaneous injection of inclisiran sodium (300 mg) on Day 1 on top of background rosuvastatin and ezetimibe, followed by a second scheduled dose at Day 90. A routine clinical safety and lipid panel at Month 3 (prior to the second injection) confirmed early therapeutic response, with LDL-C declining to 58 mg/dL (a 59.1% reduction from pre-inclisiran baseline). At Month 6 (90 days following his second injection), the follow-up lipid profile demonstrated a total cholesterol of 112 mg/dL, a drop in LDL-C to 28 mg/dL (an 80.2% absolute reduction from baseline), triglycerides of 118 mg/dL, and an HDL-C of 46 mg/dL (Table 1). HIV-1 RNA remained fully suppressed (<20 copies/mL) with a stable CD4+ count (702 cells/mm^3^). No myalgias, transaminase elevations, or injection-site reactions were observed.

### 2.2. Case 2: Severe Statin Intolerance and Primary Prevention Failure

A 58-year-old male with a 12-year history of HIV infection on a stable ART regimen of dolutegravir and lamivudine (DTG/3TC) presented with severe, unmanaged hypercholesterolemia. His plasma HIV-1 RNA was below the limit of quantification (<20 copies/mL), and his CD4+ count was 540 cells/mm^3^. The patient had an estimated 10-year ASCVD risk score of 11.2%, placing him in a high-risk primary prevention category augmented by HIV as an independent cardiovascular risk-enhancing factor. His lipid panel off lipid-lowering therapy demonstrated a total cholesterol of 318 mg/dL, an LDL-C of 194 mg/dL, triglycerides of 185 mg/dL, and an HDL-C of 48 mg/dL (Table 1). Secondary causes of severe hypercholesterolemia and muscle pathology—including overt or subclinical hypothyroidism (normal TSH and free T4), nephrotic syndrome, and chronic renal impairment—were ruled out.

Prior attempts to establish statin therapy had consistently failed due to muscular adverse effects. Rosuvastatin (10 mg daily) had induced severe, debilitating proximal bilateral muscle pain and weakness within 3 weeks, accompanied by a serum creatine kinase (CK) elevation exceeding 4 times the upper limit of normal, necessitating discontinuation. Subsequent re-challenges with low-dose atorvastatin (10 mg daily) and pravastatin (20 mg daily) reproduced severe muscle pain, establishing complete statin intolerance. Ezetimibe monotherapy (10 mg daily) was subsequently trialed but produced an inadequate 12% reduction in LDL-C, leaving his LDL-C elevated at 171 mg/dL.

In line with European regulatory authorizations for statin-intolerant patients requiring substantial LDL-C lowering, inclisiran was initiated as monotherapy. Subcutaneous inclisiran sodium (300 mg) was administered on Day 1 and Day 90. At Month 3, his LDL-C decreased to 91 mg/dL. At Month 6, his follow-up lipid profile demonstrated total cholesterol of 168 mg/dL, LDL-C of 88 mg/dL (a 54.6% reduction from his untreated baseline), triglycerides of 142 mg/dL, and HDL-C of 51 mg/dL (Table 1). The patient reported no muscle soreness, physical limitations, or adverse events throughout treatment. Serum CK and liver transaminases remained strictly within normal reference ranges, while complete HIV virological suppression and CD4+ T-cell stability were maintained.

## 3. Discussion

The clinical scenarios presented above demonstrate the successful real-world application of inclisiran to address the distinct challenges of accelerated cardiovascular risk in PWH. In both cases, the initiation of this hepatic-targeted siRNA molecule resulted in profound, durable reductions in atherogenic lipid fractions without altering virological suppression or inducing drug toxicities.

The rationale for aggressive lipid optimization in PWH has been solidified by modern trial evidence [10]. The REPRIEVE trial definitively demonstrated that statin therapy protects PWH against ischemic events, largely by reducing subclinical noncalcified coronary plaque [10]. However, as illustrated by Case 1, aggressive secondary prevention targets (<55 mg/dL) are frequently unattainable with statin/ezetimibe combinations alone, particularly when antiretroviral regimens or underlying genetic susceptibilities drive persistent dyslipidaemia. Conversely, Case 2 illustrates the barrier of statin intolerance in high-risk primary prevention, where reproducible muscle pain and biochemical elevations preclude standard first-line therapies.

Targeting the PCSK9 pathway in HIV-associated dyslipidaemia was initially validated by monoclonal antibodies (mAbs) [14]. The landmark phase 3 BEIJERINCK trial demonstrated that evolocumab (420 mg monthly) was well tolerated and achieved a 56.9% placebo-corrected reduction in LDL-C among PWH on maximally tolerated statin therapy, with significant parallel decreases in apolipoprotein B and lipoprotein(a), while recent federated database analyses have linked PCSK9-targeted strategies with significantly lower odds of all-cause mortality and heart failure [15,16]. While monoclonal antibodies bind circulating extracellular PCSK9 protein requiring biweekly or monthly subcutaneous self-injections, inclisiran acts intracellularly upstream via RNA interference [14]. By complexing with the hepatic RNA-induced silencing complex (RISC), inclisiran catalytically cleaves PCSK9 mRNA, halting protein translation entirely [14]. Its infrequent, twice-yearly healthcare provider-administered dosing schedule (following an initial and 3-month loading dose) directly resolves patient-level adherence hurdles and eliminates pharmacokinetic interference with antiretroviral agents [14]. In our clinical practice, the decision to utilize inclisiran rather than monoclonal antibodies was driven primarily by adherence optimization and dosing logistics. By shifting administration to an infrequent, twice-yearly healthcare provider-administered model (at Day 1, Day 90, and every 6 months thereafter), inclisiran effectively eliminates daily pill burden and at-home injection non-compliance.

Our clinical observations correlate strongly with recent aggregated data from large clinical trials and real-world registries. Large-scale pooled meta-analyses, such as those by Basit et al. involving 5016 patients and Cheng et al. encompassing 4947 patients, have demonstrated that inclisiran consistently abates circulating LDL-C levels by 46.95% to 50.42%, while simultaneously depressing total circulating PCSK9 levels by over 70% and apolipoprotein B (apo B) by up to 41.47% [17,18]. Furthermore, Khalil et al. reported a substantial, reproducible placebo-corrected mean reduction of 51.25% for LDL-C and an associated 21.77% reduction in Lipoprotein(a), matching the lipid shifts observed in our first patient [19].

The surrogate biomarker efficacy observed in our patients aligns with the pivotal phase 3 ORION program (ORION-9, ORION-10, and ORION-11), which demonstrated an approximate 50% placebo-corrected LDL-C reduction across primary and secondary prevention cohorts. While dedicated cardiovascular outcome trials (ORION-4 and VICTORION-2 Prevent) remain ongoing to quantify hard event reductions, real-world registries consistently replicate these lipid-lowering parameters [20,21,22]. The prospective Italian CHOLINET registry (n = 659) reported a median 51% LDL-C reduction at 3 months, which escalated to 56% at 9 months, particularly among patients on a background of statin and ezetimibe therapy [23]. A systematic review of real-world cohorts by Alaíz et al. confirmed an average real-world LDL-C lowering of 42.77% across diverse practice settings, with greater efficacy seen in combination cohorts (45.67%) versus monotherapy cohorts (37.53%) [24]. Crucially for our second case, the phase 3 VICTORION-Mono randomized clinical trial proved that in primary prevention populations lacking background lipid therapies, inclisiran monotherapy was significantly superior to both placebo and ezetimibe, reducing LDL-C by 46.5% at Day 150 [25]. This confirms that the absence of concurrent statin therapy does not blunt the intracellular gene-silencing efficiency of the siRNA molecule.

The differential magnitude of response observed between our two patients (80.2% in Case 1 vs. 54.6% in Case 2) highlights the mechanistic synergy of combination lipid-lowering therapy. Oral agents, such as statins and ezetimibe, activate sterol regulatory element-binding protein 2 (SREBP-2) to upregulate hepatic LDL receptor (LDLR) expression, while inclisiran prevents PCSK9-mediated LDLR lysosomal degradation [15]. This dual mechanism maximizes cell-surface receptor recycling and clearance of circulating atherogenic particles. In contrast, in the setting of complete statin intolerance (Case 2), inclisiran monotherapy achieved a 54.6% reduction, closely mirroring the 46.5% reduction reported in the phase 3 VICTORION-Mono trial for treatment-naïve patients.

From a safety perspective, inclisiran was well tolerated in both individuals. Neither patient experienced injection-site reactions, muscle discomfort, nor transaminase elevations, and both maintained stable CD4+ cell counts and complete virological suppression throughout follow-up. Shifting the treatment model to an infrequent, twice-yearly healthcare provider-administered regimen directly resolves daily oral pill fatigue and at-home self-injection non-adherence, offering a dependable approach for lipid management in PWH.

## 4. Conclusions

These two clinical cases illustrate that inclisiran can achieve substantial surrogate LDL-C reductions in people living with HIV across both secondary prevention and primary prevention with confirmed statin intolerance. By providing a provider-administered regimen every 6 months, inclisiran circumvents oral adherence fatigue and lacks pharmacokinetic interactions with antiretroviral therapy. While dedicated outcome trials are necessary to quantify long-term cardiovascular event reduction, our preliminary observations indicate that PWH with severe or refractory dyslipidaemia tolerate inclisiran well and achieve anticipated lipid responses without compromising virological suppression.

## Figures and Tables

**Table 1:** Lipid profile of PWH at baseline and 6 months after initiation of Inclisiran.

Lipid Parameter	Case 1		Case 2	
	Baseline (Oral LLT)	Month 6 (With Inclisiran)	Baseline (Oral LLT)	Month 6 (With Inclisiran)
Total Cholesterol	224 mg/dL	112 mg/dL	318 mg/dL	168 mg/dL
LDL-C	142 mg/dL	28 mg/dL	194 mg/dL	88 mg/dL
Triglycerides	158 mg/dL	118 mg/dL	185 mg/dL	142 mg/dL
HDL-C	41 mg/dL	46 mg/dL	48 mg/dL	51 mg/dL

## Data Availability

The research data are available after apply to the corresponding author due to privacy concerns.
